# Readiness of Medical Students and Budding Doctors About the LGBTQ+ Community: A Cross-Sectional Study From India

**DOI:** 10.7759/cureus.55177

**Published:** 2024-02-28

**Authors:** Paras Waghmare, Anjali Mediboina, Harshita Harshita, Ujwala U Ukey, Piyush Kherde

**Affiliations:** 1 Department of Community Medicine, Government Medical College and Hospital, Nagpur, Nagpur, IND; 2 Department of Community Medicine, Alluri Sitarama Raju Academy of Medical Sciences, Eluru, IND; 3 Department of Community Medicine, All India Institutes of Medical Sciences (AIIMS) Rishikesh, Rishikesh, IND; 4 Department of Physiology, Government Medical College and Hospital, Nagpur, Nagpur, IND

**Keywords:** health, india, medical students, knowledge and attitudes, lgbtq+

## Abstract

Introduction: The Lesbian, Gay, Bisexual, Transgender, and Queer or Questioning (LGBTQ) community has always faced discrimination across the globe. Due to a lack of information in medical curricula and no training or sensitization of doctors towards their health needs, there are numerous health disparities faced by this community. Negative attitudes and inadequate knowledge may also cause students to feel hesitant to treat patients in the community. The present study thus aims to evaluate the attitudes and knowledge of medical students and interns towards LGBTQ+.

Methodology: The present study is a cross-sectional observational study where a self-administered questionnaire was shared with the medical students of India via an online platform. The collected data was then analyzed using Microsoft Excel STATA version 14.0 (StataCorp LLC, Texas, USA).

Results: A total of 790 responses were analyzed. 67.2% of respondents had an overall positive attitude, with students under the age of 20, female students, and medical students being more likely to have a positive attitude towards the community. 60.7% of the respondents had satisfactory knowledge, with interns being more likely to have more knowledge than students. The respondents with better knowledge were more likely to have a positive attitude.

Conclusion: The study participants had satisfactory knowledge, and the majority had a positive attitude towards the LGBTQ+ community. However, to clear misconceptions regarding the LGBTQ+ community, the medical curriculum must be updated to include more information and provide proper training and sensitization in order to ensure optimum healthcare for all, regardless of sexual orientation and identity.

## Introduction

LGBTQ+ stands for lesbian, gay, bisexual, transgender, and queer or questioning. The ‘+’ signifies that one acronym cannot capture everyone’s experience of their gender identity, expression, and/or sexual orientation [1].

LGBTQ+ individuals face social stigma and a lack of gender-inclusive healthcare and are at a greater risk of health disparities; for example, the LGBTQ+ community is one of the 12 groups least represented in health equity in India. There are higher incidences of psychiatric issues [2] like depression and anxiety, as well as drug abuse and suicides, among the community [3,4]. Aside from mental health impacts, numerous studies have shown that there are impacts on physical health as well. The incidence of sexually transmitted diseases, breast and cervical cancer, obesity, and eating disorders, among other health problems, is higher in LGBTQ+ individuals [5-7].

Due to a lack of information in medical curricula and no training or sensitization of doctors towards health needs, there are numerous health disparities faced by this community. The community largely remains unnoticed due to the overall stigmatization of the topic per se, despite the discrimination faced by LGBTQ+ individuals in all walks of life. Negative attitudes and inadequate knowledge make medical students feel hesitant to treat patients in the community. These health disparities could be attributed to a lack of knowledge regarding the community. Literature has shown that over half of medical school curricula include little or no information about gay and lesbian individuals [8]. In India, there is no direct information regarding gender-affirming surgeries or gender dysphoria. Further ahead, on account of the stigma and stereotypes surrounding the community, many doctors would try to avoid touching their patients and would even try to avoid providing necessary treatment [9]. Thus, it is imperative to address the special health needs of the third gender, which demands a baseline survey of the knowledge and attitude of future medical practitioners about LGBTQ+.

Considering this background, the current study was conducted with the objectives of estimating the knowledge and attitudes of medical students and interns regarding LGBTQ+ health, creating awareness among the respondents regarding the health disparities being faced, and contributing to the medical literature regarding the LGBTQ+ community.

## Materials and methods

Study type and setting

It was a descriptive observation study (cross-sectional) done using a questionnaire conducted among undergraduate medical students across India. India is a developing country located in the Southeast Asian region. The study was conducted among Indian medical students from different states. Medical education in this country does not cover the health aspects of LGBTQ+ separately in its syllabus, though it is a part of different subjects during the MBBS course.

Study duration and population

The study was carried out over a period of six months. All undergraduate medical students studying in MBBS or doing their internship were invited to participate in this cross-sectional survey.

Study tool

It was a predesigned and pre-structured questionnaire that included various open-ended and closed-ended questions totaling 25 in number. The study instrument thus developed was thoroughly checked for validity of content by subject experts who were medical personnel and had received training on the topic. It was piloted and pre-tested for feasibility with 20 students (from the same medical college) before actual use. Based on the results of the pilot, the required modifications to the questionnaire were made to prepare the final version (Appendix).

Data collection

Data were collected via an online survey with the aid of a self-administered questionnaire that was prepared in Google Form in English. The link to the Google form was sent through social media like WhatsApp groups, Instagram, Telegram, and e-mails. In this study, the questionnaire was shared with the contacts of the researchers, who were requested to share the same with their contacts, who further shared the questionnaire with their contacts. This was continued for the entire data collection period.

Sampling technique and sample size

A non-probability convenience sampling technique was used to collect the responses. The sample size was calculated with the aid of STATA version 14.0 (StataCorp LLC, Texas, USA). Considering the high knowledge of 71.9% of medical students based on the reference study of Wahlen et al. [10] at a 95% confidence interval and with a relative precision of 5%, the minimum estimated sample to be covered was 628.

Ethical aspects and confidentiality

The institutional ethics committee (IEC) of Government Medical College Nagpur (2314EC/PHARMAC/GMC/NGP) approved the study before its commencement. The questionnaire had a brief description of the nature and purpose of the study. Participation in the study was entirely voluntary. The study tool had an informed consent form appended to it, which marked the response in the form of yes or no. Further, participants' consent was also implied by their voluntary completion of the questionnaire. Due care was taken to maintain the anonymity and confidentiality of the responses received.

Data analysis

Data in Microsoft Office Excel spreadsheets (Microsoft® Corp., Redmond, WA) was coded and cleaned. The duplicate responses were removed. Descriptive data were expressed as percentages and means. Statistical analysis was done with the help of the software STATA. The chi-square test was used to calculate the association of attitude and knowledge with the independent variables. A bivariable analysis was performed to find an association between satisfactory knowledge scores and variables like age, gender, and occupation (student or intern) of the study participants. The multivariable logistic regression analysis for these variables was also carried out to calculate the adjusted odds ratio and 95% CI. A p-value of < 0.05 was considered to be statistically significant.

## Results

Sociodemographic data

A total of 800 medical students responded to the questionnaire circulated, out of which seven did not agree to participate in the study, and responses from three were not eligible for the survey. Though the minimum calculated sample size was 628, the researchers considered all the 789 responses. In the present study, 486 (61.5%) responses were received from female students, and the remaining 303 (38.4%) were from male students. Fifty-seven percent of the participants were less than 20 years old, and the other 43% were more than equal to 20 years of age. The details of the general characteristics are shown in Table 1.

**Table 1:** General characteristics of study participants

Characteristic	Number	Percentage
Age in years		
18	49	6.2%
19	174	22.02%
20	224	28.35%
21	168	21.26%
22	101	12.78%
23	54	6.83%
>24	19	2.4%
Gender		
Male	304	38.48%
Female	486	61.52%
Academic year		
1st MBBS	284	35.94%
2ndMBBS	234	29.62%
3rd MBBS	104	13.16%
4th MBBS	110	13.92%
Internship	61	7.72%

In the present study, responses were received from different states of the country. The state-wise distribution is shown in Table 2.

**Table 2:** State-wise numerical representation of responses received

Sr.	State name	No. of response
1	Andhra Pradesh	244
2	Assam	1
3	Chhattisgarh	2
4	Daman and Diu	1
5	Delhi	50
6	Uttarakhand	161
7	Goa	2
8	Gujrat	3
9	Himachal Pradesh	5
10	Jammu & Kashmir	1
11	Karnataka	30
12	Kerala	4
13	Madhya Pradesh	10
14	Maharashtra	211
15	Orissa	1
16	Punjab	6
17	Rajasthan	6
18	Tamil Nadu	22
19	Telangana	9
20	Uttar Pradesh	19
21	West Bengal	2
Total		790

Attitudes towards the LGBTQ+ community

In the present study, the attitude of the medical students towards LGBTQ+ was assessed with the help of 10 closed-ended questions. Most of the students had a positive attitude towards the LGBTQ+ community. The least stigmatized attitude was towards the statement “LGBTQ+ patients deserve the same level of quality care from medical institutions as heterosexual patients,” with 85% of the respondents choosing “strongly agree.” The statement with the most neutral responses was “I believe my medical training adequately addressed the health needs of the LGBTQ+ population,” for which 28% of the respondents were neutral. “Sex and gender have the same meaning” was the most stigmatized statement, with 8% of the participants strongly agreeing with it. Table 3 tabulates the distribution of attitudes towards LGBTQ based on age, gender, and profession.

**Table 3:** Distribution in attitude towards LGBTQ in age, gender, and profession

Attitude towards LGBTQ	Age distribution		Gender distribution		Profession distribution	
	>20	<20	Male	Female	Intern	Students
Negative	40	55	58	37	6	89
Positive	301	393	245	449	55	693
Chi-square	0.05		23.42		0.30	
P-value	0.8152		<0.0001		0.5818	

Knowledge regarding health of the LGBTQ+ community

The most controversial statement was “LGBTQ+ people can be identified by certain mannerisms and characteristics,” with 51% of the participants opting for "true" and 49% for "false."

The statement for which the maximum number of participants gave the correct answer was “LGBTQ+ patients may present with signs of depression due to a lack of social acceptance,” with 98.48% of respondents choosing “true.” The statement for which the maximum number of respondents gave the wrong answer was “Bisexual and lesbian women are at a greater risk of developing obesity than heterosexual women,” with only 18.6% choosing “true.”

The maximum difference between responses of clinically experienced and clinically inexperienced groups was seen for the statement, “Sexual/gender minorities experience greater risk for sexually transmitted infections than heterosexuals and gender majorities.” 70% of interns and year four students, while only 50% of years 1, 2, and 3 students responded correctly to this statement. Age, gender, and profession-wise distribution in knowledge and assessment of it with regard to LGBTQ are tabulated in Tables 4-5.

**Table 4:** Distribution in knowledge towards LGBTQ in age, gender, and profession

Knowledge towards LGBTQ	Age distribution		Gender distribution		Profession distribution	
	>20	<20	Male	Female	Intern	Students
Satisfactory	518	47	264	39	59	646
Non-satisfactory	187	37	441	45	2	82
Chi-square	11.3359		2.5597		3.773	
P-value	0.001		0.123		0.031	

**Table 5:** Assessment of knowledge of study participants regarding LGBTQ+ health in India

Statement	Correct response number	Incorrect response number
LGBTQ+ people can be identified by certain mannerisms and characteristics (FALSE)	386	407
LGBTQ+ patients do not seek medical treatment as early as heterosexuals because of fear of discrimination (TRUE)	708	85
LGBTQ+ individuals are more likely to be victims of violent crime (TRUE)	629	184
LGBTQ+ patients may present with signs of depression due to lack of social acceptance (TRUE)	781	12
In order to be considered as a transgender, a person must have undergone a sexual reassignment surgery (FALSE)	679	114
Transgender men do not need to undergo screening tests for cervical cancer (FALSE)	652	141
A transgender person must be addressed using pronouns of preferred gender rather than biological sex (TRUE)	640	153
Rates of domestic violence are less in coupled gay male households (FALSE)	368	425
Bisexual and lesbian women are at a greater risk of developing obesity than heterosexual women (TRUE)	148	645
Sexual/gender minorities experience greater risk for sexually transmitted infections than heterosexuals and gender majorities (TRUE)	431	362

Views and opinion on the health of LGBTQ+ community

In the questionnaire, with the assessment of attitude and knowledge, we also added one open-ended question, i.e., “Your views on the health of the LGBTQ+ community." Out of 789 responses, we have 300 who shared their views and opinions on the health of LGBTQ individuals. They also expressed their views on "LGBTQ and society," as many people in society do not accept people who belong to the LGBTQ community. We received a wide range of responses. Important ones are summarized ahead. Some respondents expressed that “LGBTQ is a community that has been recognized recently due to social awareness. People are now ready to acknowledge their existence and respect them with equal dignity. This is all because of those few LGBTQ people who stood up for themselves and encouraged others to do the same. This has created a major change in the thought process of a person with any kind of social activity. But this change needs to spread to other platforms, too, like schools, courts, medical colleges, etc. Our current system needs a major reform until LGBTQ is treated the same as heterosexuality and they no longer feel left out of society.” Few respondents even expressed that many people consider LGBTQ as a disease, but the fact of their existence should be accepted, and these communities should also be provided with an opportunity for a respectful livelihood. Health education on the topic in the general population right from schooling can also increase its awareness and eventually acceptance.

Some students were of the opinion that LGBTQ+ people experience a number of health disparities. They’re at higher risk of certain conditions, have less access to health care, and have worse health outcomes. These disparities are seen in the areas of behavioral health, physical health, and access to care. They deserve the same kind of medical care. Lack of financial independence and social norms make appropriate health facilities inaccessible to them. The LGBTQ health examination should be included in mainstream medical education, as there can be several misconceptions due to a lack of awareness rather than insensitivity. Their health problems are different and probably lie more on the side of venereal diseases because of their lifestyle choices, and this can be a dilemma during treatment because preventing the disease comes at the cost of having to scrutinize their lifestyle choices. As humans, they also require equal and fair treatment without judgment. So, discrimination against their community in terms of healthcare is strongly unacceptable.

Very few, i.e., two of the respondents, were of the opinion that "the LGBTQ is abnormal and is against the law of nature. They are morally and ethically unacceptable." There are higher chances of STDs among the LGBTQ+ community.

However, most of the responses pointed towards the need to accept the community as a human being. There was a need for increasing knowledge about this gender, as expressed by many study participants. In addition, lack of access to appropriate health facilities due to financial dependence and social norms increases the problem of violence against these minorities. The need for adequate health facilities to be provided for them was also emphasized by many.

## Discussion

Studies from Indian settings focusing on the LGBTQ+ community are few in number, though numerous studies on the topic have been carried out globally [1-8]. Further ahead, there is a dearth of literature specifically assessing the knowledge and attitudes of medical students towards this community in India. The study in Chhattisgarh, India, by Nagrale et al. covered a sample of 200 participants, which included 61.5% female and 38.5% male students [11]. Hence, the present study, involving responses from 789 participants, is one of the unique ones that has estimated the attitudes and knowledge of medical undergraduates across India about this unexplored area. Whereas the study in Nepal by Pandey et al. had a greater number of male respondents [12]. This difference could be because of the higher ratio of female students enrolled in medical colleges in India [13], and also because the present study was an online survey, and probably more female respondents completed the questionnaire.

The majority of the participants were female students. Approximately more than half of the respondents were less than 20 years of age. The study covered responses from various provinces of the country, thus representing medical students belonging to a multitude of cultural and social backgrounds. Other studies by Nagrale et al. in India, Pandey et al. in Nepal, and Khalaf et al. in Malaysia were carried out only in a single set-up [11,12,14]. The fact that the current study had participants from different medical colleges across the country gives a better picture of the scenario.

The correct knowledge about the LGBT community and health issues related to them was present in 38.35% of medical students who responded to the study. This observation is coherent with the findings as reposted by Banwari et al. [15]. However, the responses in the study by Dunjić-Kostić et al. reflected better knowledge [16].

In the present study, attitudes towards the LGBTQ+ community are overall positive. For the statement “Sex and gender have the same meaning,” the mean score was 2.4 for students in years 1, 2, and 3, and 2.3 for year four students and interns; however, the overall percentage of people who agreed or were neutral to the statement was 42%. This implies that there are still a good number of students who are not aware of the difference between sex and gender. For statements such as “homosexuality is a disease and requires a cure," “one cannot be bisexual; it is merely experimenting,” and “sex-change operations are morally wrong” overall, majority of the respondents, i.e., 84%, 77%, and 65.8%, respectively, disagreed with the statements. Thus, it can be inferred that a majority of the students have an overall positive attitude towards the LGBTQ+ community. This observation coincides with the study of Pandey et al. conducted in Nepal wherein majority also had a positive attitude towards LGBTQ community. These observations indicate that medical students of the era are giving more importance to the gender equality and accepting the existence of genders other than male and female. Because of this, LGBTQ community will have the better acceptance of the health services which may result in their better health status. There was no significant correlation between level of clinical exposure and such comparison is not made by previous study. The reason for no statistical difference could be on account of the fact that the positive attitude is in general and not influenced by gender and age.

The main limitation of this study is that, with India being culturally and sexually conservative, respondents may feel less comfortable expressing their views on sexuality-related issues and might not have been open to all the questions. Another limitation is that, though the study mentions participation from across the country, there is variation in the number of responses from different states, and the original state of residence was not taken into account. The study has some limitations inherent in a cross-sectional study, such as that it gives information about that cross-section of time and place only, and there is no follow-up. The strength of the study is that it included participants from all over India. The online nature of the study has made it possible to reach participants in different states. Further ahead, anonymity could also be maintained.

## Conclusions

Medical curricula in India have little to no information regarding the LGBTQ+ community. Despite the stigmatization of the community, the present study found overall positive attitudes and fair knowledge among medical students in India. The study found no correlation between the level of clinical exposure and attitude towards the community.

LGBTQ+ patients’ health needs and disparities must be included in the medical curriculum to spread awareness and remove prejudice towards the community. More information and training are required to ensure that all citizens of India, regardless of sexual orientation and identity, receive optimum healthcare.

## Appendices

Study questionnaire

**Table 6:** Study questionnaire for the assessment of attitude and knowledge towards LGBTQ+ community

Section A (consent)	Response						
	Yes			No			
I, the undersigned, am voluntarily willing to participate as a study subject in this research study. I have been informed in detail regarding the investigative procedures and techniques involved in this study in the language I understand. I have no objection whatsoever to the results of the study being published for the advancement of medical knowledge, as long as confidentiality is maintained and my identity is not revealed anywhere. I have fully understood the aforesaid and willingly give my consent for the same.							
Section B (general information)	Response						
Age (in years)							
Gender	Male	female			Other		
Name of College:							
State of College:							
Year of Profession	I MBBS	II MBBS	III (Part 1) MBBS			III (Part 2) MBBS	Intern

Section B: Attitude towards the LGBTQ+ community. Please answer the following questions on a scale of 1 to 5 Strongly Agree, Agree, Neutral, Disagree, Strongly Disagree.							
Sr. No.	Questions	Response					
		1		2	3	4	5
1	Sex and gender have the same meaning.						
2	LGBTQ+ patients deserve the same level of quality care from medical institutions as heterosexual patients.						
3	I believe my medical training adequately addressed the health needs of the LGBTQ+ population.						
4	Homosexuality is a disease and requires a "cure".						
5	Homosexual individuals are incapable of being good parents.						
6	One cannot be bisexual or attracted to both males and females; it is merely experimenting.						
7	Just like homosexuality and heterosexuality, bisexuality is also a stable sexual orientation.						
8	I avoid transgenders when I see them.						
9	"Sex-change" operations are morally wrong.						
10	I would offer treatment and medical care for a patient if I were aware they identified as LGBTQ+.						
Section C: Knowledge towards the LGBTQ+ community. Please answer the following questions in TRUE or FALSE.							
Sr. No.	Questions		Responses				
			True			False	
1	LGBTQ+ patients do not seek medical treatment as early as heterosexuals because of fear of discrimination.						
2	LGBTQ+ individuals are more likely to be victims of violent crime.						
3	LGBTQ+ patients may present with signs of depression due to lack of social acceptance.						
4	In order to be considered as transgender, a person must have undergone a sexual reassignment surgery.						
5	Transgender men do not need to undergo screening tests for cervical cancer.						
6	A transgender person must be addressed using pronouns of preferred gender rather than biological sex.						
7	LGBTQ+ people can be identified by certain mannerisms and characteristics.						
8	Rates of domestic violence are less in coupled gay male households.						
9	Bisexual and lesbian women are at risk of developing obesity than heterosexual women.						
10	Sexual/gender minorities experience greater risk for sexually transmitted infections than heterosexuals and gender majorities.						
